# Cytomegalovirus retinitis and antiretroviral treatment: A fifteen year experience

**DOI:** 10.4102/sajhivmed.v23i1.1322

**Published:** 2022-03-08

**Authors:** Serisha Jay Narain, Linda Visser, Wilbert Sibanda

**Affiliations:** 1 Department of Ophthalmology, Faculty of Health Sciences, University of KwaZulu-Natal, Durban, South Africa; 2 Department of Ophthalmology, Faculty of Health Science, Stellenbosch University, Cape Town, South Africa; 3 Biostatistics Unit, Faculty of Health Sciences Management, Nelson Mandela University, Gqeberha, South Africa

**Keywords:** cytomegalovirus retinitis, opportunistic infection, ocular, acquired immunodeficiency disease, human immunodeficiency virus, antiretroviral therapy

## Abstract

**Background:**

South Africa’s public antiretroviral treatment (ART) programme has undergone progressive changes since its introduction in 2004. The effect of this on the burden of the AIDS-defining opportunistic infection, cytomegalovirus retinitis (CMVR), in SA, has not been fully appreciated.

**Objectives:**

To determine the effect of ART availability in the public sector of SA on the trend in the number of cases of newly diagnosed CMVR over time.

**Methods:**

This is a retrospective study from 01 November 2002 to 31 August 2017 that took place at a tertiary hospital in the KwaZulu-Natal (KZN) province.

**Results:**

A total of 383 participants were included in the study, with 60.1% being female and 94% of black African origin. The mean age of patients was 34.08 years (SD ± 7.24). A linear trend model suggested an overall linear decrease in the number of new cases of CMVR per year (*R*^2^ of 0.67). The average number of new cases of CMVR per year prior to ART being available to all persons living with HIV (PLWH) with a CD4+ ≤ 350 cells/μL and after was 34 and 13, respectively, and the difference (61.76%) between these values was statistically significant, *P* = 0.001. The median CD4+ count at diagnosis of CMVR was 22 (interquartile range: 9–51.25) cells/μL. An overall 51% of patients in this study were on ART at diagnosis of CMVR. There was a higher proportion of patients on ART ≤ 6 months (63.3%), compared with those on ART > 6 months (36.7%), and the difference was statistically significant, *P* < 0.01.

**Conclusion:**

ART has resulted in a decrease in the burden of CMVR on ophthalmic services for many in KZN, particularly following the introduction of ART for all PLWH with a CD4 ≤ 350 cells/μL.

## Introduction

In 2018, it was estimated that 21 million of a global total of 38 million persons living with HIV (PLWH) live in Eastern and Southern Africa.^1^ With South Africa (SA) being the epicentre of HIV, the estimated numbers of PLWH have increased from 3.8 million in 2002^2^ to 7.64 million in 2020.^3^ Antiretroviral treatment (ART) has been used in the United States (US) and Europe since 1996;^4,5,6,7^ however, the public sector in SA was only able to access therapy from 2004, and then only for those with a CD4+ count ≤ 200 cells/µL.^8,9,10^ Despite the delayed start, SA expanded access to ART in 2012 and 2015, when treatment was offered to all with a CD4+ count of ≤ 350 cells/μL and 500 cells/µL respectively.^10,11,12^ In 2015, SA adopted the 90-90-90% targets of the Joint United Nations Programme on AIDS (UNAIDS). These aimed to ensure that 90% of the population were aware of their HIV status, 90% of those living with HIV would be on ART and 90% of the latter be virally suppressed by 2020.^8,9^ In 2016, the ‘Universal Test and Treat’ (UTT) programme was implemented in SA, and ART was offered to all PLWH regardless of their CD4+ level.^13^ South Africa now has the world’s largest ART programme,^8,9^ and numbers on ART have been increasing from 45 500 in 2004 to 4.12 million in 2017.^9,14^ Approximately, 30% of this number live in KwaZulu-Natal (KZN), a province of SA,^9^ with eThekwini being the largest city. In 2018, 16.8% of the city population (3.86 million) were estimated to be HIV positive. Seventy-three per cent were believed to be on ART.^15^ These demographic changes have impacted healthcare locally and influenced the incidence of opportunistic infections linked to AIDS, in particular cytomegalovirus (CMV) retinitis (CMVR).

A 2016 review of CMV co-infection of PLWH in Africa found 100% CMV-seroprevalence in two studies of South African PLWH.^16,17,18^ Cytomegalovirus retinitis is the most common form of end-organ CMV disease of PLWH. It occurs at CD4 levels that indicate severe immune impairment, for example ≤ 100 cells/μL.^16^ Indeed, levels at or below 50 cells/μL are considered usual.^1,4,5,6,7,19,20^ Cytomegalovirus (CMV) retinitis can be bilateral or unilateral, symptomatic or asymptomatic.^7^ Presenting visual symptoms include floaters, scotomas and decreased visual acuity.^7^ The diagnosis of CMVR is clinical (eye examination and fundoscopy) which is also the ‘gold standard’ for CMVR research.^4,5,6,7,19,20,21^ The main differential diagnosis is progressive outer retinal necrosis secondary to varicella zoster virus (VZV). However, this condition occurs at higher CD4+ counts and has a different clinical presentation.^1^

Untreated CMVR progresses to permanent visual loss and recurs if anti-CMV therapy is discontinued prior to CD4+ recovery on ART, that is, an increase in the CD4+ count to ≥ 100 cells/µL and its persistent upward trajectory on ART.^4,5,6,7,19^ However, CMV-specific immune recovery can take up to six months.^5,7,22^ Anti-CMV therapy is always the recommended initial therapy in patients who are ART naive. Followed by ART after 2 weeks on anti-CMV therapy: this is to reduce the risk of a CMV-related immune recovery inflammatory syndrome (IRIS).^21^

With regard to CMVR, IRIS may take two forms. The first is *immune recovery uveitis* (IRU), that is, an increase in or new onset of anterior uveitis or vitritis following the initiation of ART, a condition commonly associated with a quiescent retinitis.^4,5,7,23^ A case definition for the second form, *immune recovery retinitis* (IRR), has been proposed. This condition can present as an increased *rate* of new-onset CMVR (unmasking IRIS) or a worsening (paradoxical IRIS) of existing CMVR.^23^ IRR has subsequently been shown to be rare.^5^ Furthermore, IRU is on the decline in the ART era.^5^ Anti-CMV therapy may be administered orally, intravenously or intravitreally. Since 1996, there has been a significant decline in CMVR in developed countries in the ART era.^4,5,6,7^ This decline is related to access to ART prior to the development of severe immune impairment and CMVR. Furthermore, ART allows those with advanced HIV disease and asymptomatic CMVR to subsequently achieve immune recovery. Prior to this, CMVR was responsible for 90% of HIV-related visual loss and affected up to 30% of all patients with HIV in the developed world.^1^

Much of our knowledge regarding the trends in incidence rates of CMVR in the ART era originates from the CMVR subset of the US-based Longitudinal Study of Ocular Complications of AIDS (LSOCA).^5,6^ Unfortunately, studies conducted in developing countries cannot easily be compared with those of high-income regions. The study populations of low- and middle-income regions are often heterogeneous, demonstrate limited access to high-level healthcare and the incomplete recording of data, such as the CD4+ count, HIV viral load, the diagnostic workup and treatment given.^1,20^ Two significant CMVR studies have been published from SA. The first was conducted in KZN in the pre-ART era,^24^ whilst the second study was mostly centred on the ART era and was conducted in the Western Cape (WC) province.^25^ In 2017, KZN recorded the highest overall prevalence of HIV in the country (27%) and the WC the lowest (12.6%).^26^ Neither of these studies documented the trend in the number of newly diagnosed CMVR cases over time. We are yet to ascertain whether the expansion of our ART roll-out in SA has decreased the burden of CMVR. We set out to conduct a retrospective observational study of the number of new cases of CMVR in eThekwini and surrounding areas between 01 November 2002 and 31 August 2017. The primary objective of this study was to determine the effect of ART availability in the SA public sector on the trend in the number of newly diagnosed cases of CMVR over time.

## Methods

This was a retrospective chart review for the period 01 November 2002 to 31 August 2017 carried out at Inkosi Albert Luthuli Central Hospital (IALCH), a tertiary hospital that was established at eThekwini on 01 November 2002. A search for all patients presenting to the eye clinic at IALCH with the international classification of diseases-10 (ICD-10) codes H32 (chorioretinal inflammation infectious disease), B25 (CMV disease) or H30 (chorioretinal inflammation) was made using the hospital information system (HIS) at IALCH.

**FIGURE 1:**
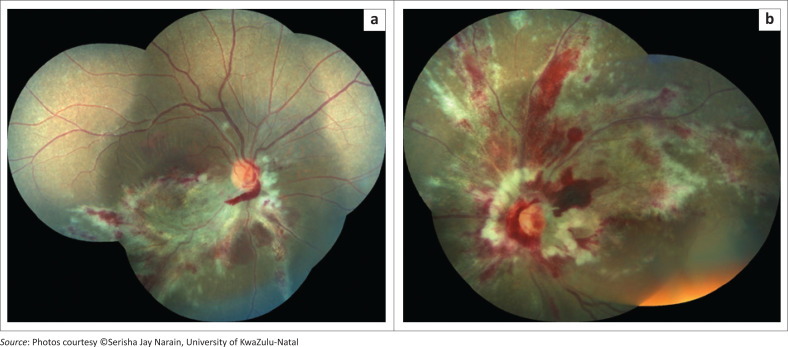
Bilateral fulminant cytomegalovirus retinitis at diagnosis.

**FIGURE 2:**
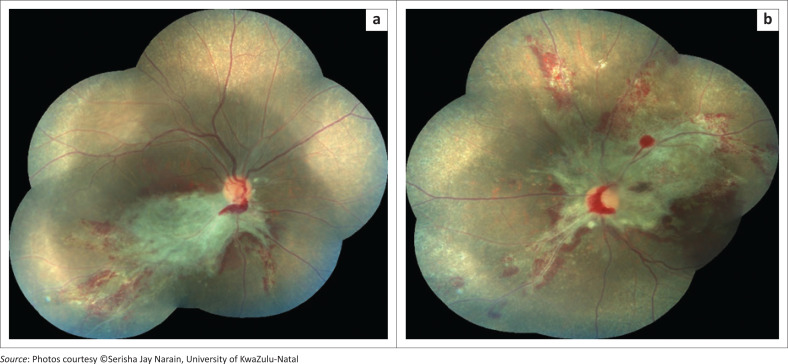
Patient from Figure 1 with bilateral cytomegalovirus retinitis after six intravitreal ganciclovir injections to both eyes.

The clinical gold standard for diagnosis of CMVR was applied: the observation of typical CMVR retinal lesions on indirect fundoscopy by an ophthalmologist.^4,6,7,19,20,21^ Typical CMVR was defined as either fulminant (brushfire), which includes perivascular wedge-shaped areas of marked retinal whitening with associated haemorrhages (Figures 1 and 2), or indolent (granular) with small opaque white dot-like lesions.^4,6,7,19,20,21^ A single ophthalmologist with considerable expertise in infectious retinitis supervised the ophthalmic team managing the participants for the duration of the study, which, in turn, improved consistency in the clinical diagnosis, management and documentation. Whilst fundus photographs were taken for the majority of participants, it was not an inclusion criterion. Vitreous fluid analysis for CMV, herpes simplex virus (HSV), VZV and toxoplasmosis, as well as syphilis serology, is only necessary in atypical retinal lesions and was, therefore, not performed for these patients with typical CMVR lesions.^4,6,7,19,20,21^

All individuals 18 years and above living with HIV and presenting with CMVR were eligible for the study. Patients who had an atypical picture at presentation or co-existent retinitis were excluded. Typically, CMVR is associated with minimal inflammation in the anterior and intermediate layers of the eye unless associated with IRU or co-existent retinitis.^4,21^ Possible CMVR-IRU in those on ART at presentation was, therefore, considered atypical and not included in this study. Patients who were diagnosed with CMVR and commenced anti-CMV treatment at another facility prior to referral were also excluded from the study, as the diagnosis was not supervised by the above-mentioned senior ophthalmologist. This was the only centre managing CMVR in the eThekwini, Ugu, Zululand, iLembe, Uthungulu and Umkhanyakude districts of KZN. Persons living with HIV who complained of visual loss were referred to a regional ophthalmic service by nurses or clinicians without the requirement of an ocular examination. They were then referred with a provisional diagnosis to our study location. This referral pattern did not change during the study period.

The variables included the patients’ date of birth, self-reported race and gender, date of diagnosis of CMVR and the CD4+ count at diagnosis of CMVR. Also recorded was mention in the clinical notes of prior tuberculosis (TB) treatment and/or a drug history of TB treatment or ART at the time of the CMVR diagnosis. For those who were on ART, a note was made if it was initiated > 6 months or ≤ 6 months prior to the CMVR diagnosis. In addition, self-reporting by the patient of ART ‘issues’ such as poor adherence to ART or changes in the regimen was noted. Patients who reported poor adherence to ART were given a referral letter to their HIV healthcare facility for further assistance. For those who were not on ART at diagnosis, a referral was made to the local HIV healthcare facility for ART initiation, after two weeks of anti-CMV treatment. The policy guidelines provided by the KZN Department of Health (DoH) defined ART as a combination of three antiretroviral drugs from more than one antiretroviral class at the time of the study.^11,12,13^

Patients were sorted according to ART availability in the KZN public health sector on their date of diagnosis of CMVR, which was based on the SA DoH ART initiation guidelines during the study period (Table 2).^10,11,12,13^ The CMVR treatment protocol in the ART era at the study site consisted of a loading dose of biweekly intravitreal ganciclovir injections for two weeks followed by weekly injections until the CD4+ count was > 60 cells/µL. Once this was achieved, patients were injected every two weeks until the CD4+ count was ≥ 100 cells/µL, after which the intravitreal injections were stopped if the patient was on ART. In the pre-ART era, the use of intravitreal ganciclovir continued indefinitely. Oral valganciclovir or foscavir was not used. Default from intravitreal anti-CMV therapy was recorded.

### Statistical analysis

In this study, continuous variables, such as age and CD4+ count, are provided as medians (interquartile range [IQR]) based on the outcome of the Kolmogorov-Smirnov test, which indicated that the data were non-parametric. Categorical variables are expressed as proportions. A *z*-test was used to compare the average number of new CMVR cases per year before and after 01 April 2012. Time series analysis was used to model the number of new cases of CMVR diagnosed per year from 01 January 2003 to 31 December 2016 at IALCH in KwaZulu-Natal. These analyses were performed using the Statistical Package for Social Sciences (Version 27.0. Armonk, NY: IBM Corp.) and Stata statistical software (Release 16. College Station, TX: StataCorp LP). The level of significance was set at *P* < 0.05.

### Ethical considerations

Ethical approval to conduct the study was obtained from the Biomedical Research Ethics Committee of the University of KwaZulu-Natal (reference number: BE579/17).

## Results

**FIGURE 3:**
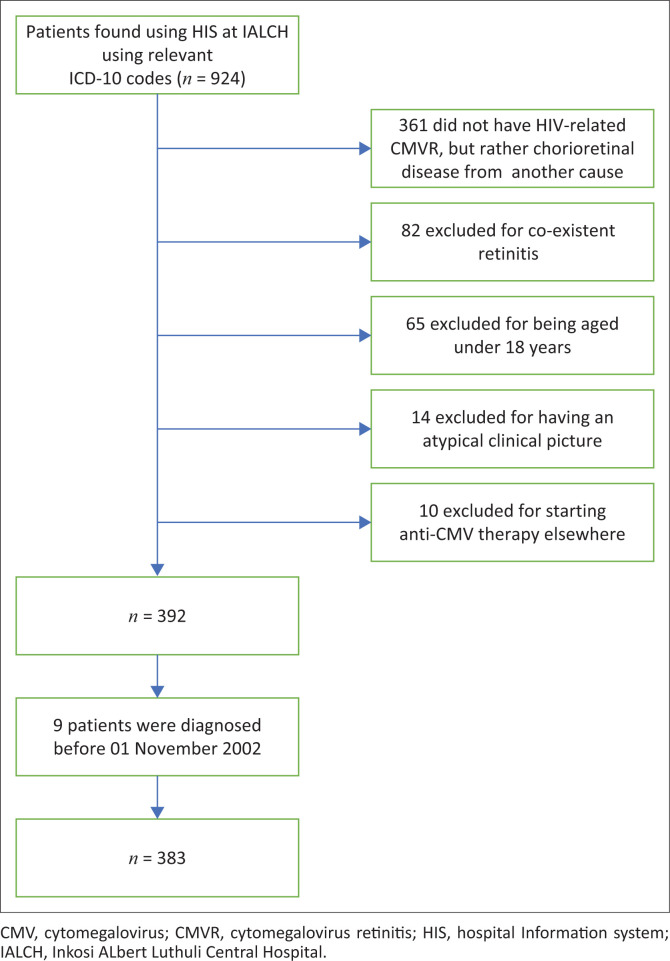
Study flow chart showing search and exclusions.

A total of 924 patients were identified using the HIS at IALCH between the period 01 November 2002 and 31 August 2017. Because of the nature of the HIS search, a large number of patients, *n* = 361, did not have HIV-associated CMVR but chorioretinal disease from an unrelated cause. Nine patients were excluded, who were diagnosed prior to 01 November 2002 when the ophthalmic unit was based at another hospital. Thereafter, a further 171 patients diagnosed with CMVR were excluded (Figure 3), with a total of 383 participants being included in this study (Table 1).

**TABLE 1:** Demographic profile of all patients.

Variable	*n*	%	95% CI
**Age-group**			
18–30	134	35	31.3–39.9
31–40	180	47	24.1–52.0
41–50	58	15.1	11.9–19.1
51–60	11	2.9	1.6–5.1
Total	383	100	-
**Gender**			
Female	230	60	55.1–64.8
Male	153	40	35.2–44.9
Total	383	100	-
**Race**			
Black African people	359	93.7	90.9–95.8
Mixed race people	4	1	0.04–2.7
Indian people	1	0.3	0.005–1.5
White African people	1	0.3	0.005–1.5
Missing	18	4.7	3.0–7.3
Total	383	100	-

CI, confidence interval.

In this study, the age range of patients was 18–69 years, with 230 (60.1%) being female and 153 (39.9%) male participants. The median age was 33.00 years (IQR: 29–38), and based on the Kolmogorov–Smirnov test, the participants’ ages were not normally distributed, *P* = 0.092. The majority of patients were of black African ethnicity, *n* = 359 (94.0 %).

**FIGURE 4:**
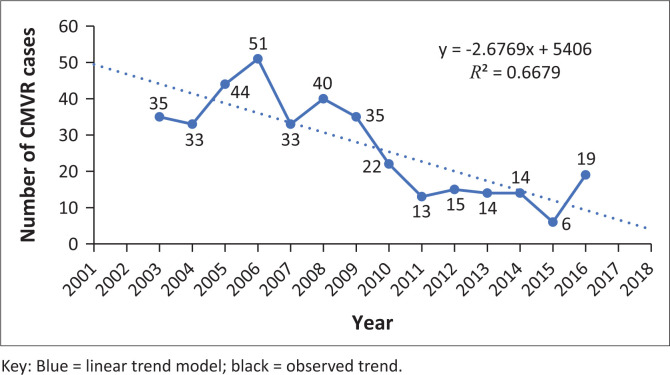
Number of new cases of cytomegalovirus retinitis diagnosed per year from 01 January 2003 to 31 December 2016 at Inkosi Albert Luthuli Hospital a large public ophthalmology healthcare centre managing the greater number of cytomegalovirus retinitis in KwaZulu-Natal, South Africa.

The number of new cases of CMVR each year was determined using the date of diagnosis, from which a trend analysis was made (Figure 4) for the period 01 January 2003 to 31 December 2016. Data collected in 2002 and 2017 were incomplete and, therefore, not included. Fitting a linear trend model (Figure 4 – blue dotted line) resulted in a coefficient of determination (*R*^2^) of 0.67. This indicated that there was an overall linear decrease in the number of new CMVR cases per year. With regard to the observed trend (Figure 4 – blue solid line), there was a 1.5× (fold) increase in the number of new cases per year between 2003 and 2006 followed by a 3.9× (fold) decrease in cases between 2006 and 2011. Thereafter, the number of new cases of CMVR diagnosed annually failed to progressively decrease with time. Notwithstanding the fact that the years 2002 and 2017 were not available, the model indicates the expected trend for the study period 2002 (36) and 2017 (6).

However, changes in ART guidelines did not necessarily correspond with the start and end of the years; we therefore determined the average number of new cases of CMVR per year with reference to the SA DoH ART initiation guidelines (Table 2). The overall average number of cases per year began to decrease during the period when ART was offered to all PLWH with a CD4+ count of ≤ 350 cells/μL. A *z*-test comparing the average number of cases per year between 01 November 2002 and 31 March 2004 (34 cases per year) and 01 April 2012 and 31 August 2017 (13 cases per year) showed the difference (61.76%) to be statistically significant (*P* < 0.001).

**TABLE 2:** Cytomegalovirus retinitis cases in correlation with antiretroviral treatment availability in eThekwini from 01 November 2002 to 31 August 2017.

ART availability	Period (years)	Total CMVR cases	CMVR cases/year (mean)
Pre-ART	01 November 2002–30 March 2004 (1.3)	47	36
< 200 cells/μL	01 April 2004–31 March 2012 (7.9)	267	34
< 350 cells/μL	01 April 2012–31 December 2014 (2.7)	39	14
< 500 cells/μL	01 January 2015–31 August 2016 (1.6)	17	11
UTT	01 September 2016–31 August 2017 (0.9)	13	14

ART, antiretroviral treatment; CMVR, cytomegalovirus retinitis; UTT, universal test and treat.

There was a significantly (*P* < 0.001; *z*-test) higher number of patients with bilateral disease, *n* = 238 (62.1%), compared with unilateral disease, *n* = 145 (37.9%). A total of 182 patients (37.5%) defaulted anti-CMV therapy. Patients with CMVR were less likely to default anti-CMVR therapy after the introduction of ART in April 2004 with a relative risk ratio of 0.548 (95% CI: 0.452–0.665; *P* = 0.001). The median CD4+ count at the date of diagnosis of CMVR was 22 (*n* = 240; IQR: 9-51.25) cells/μL. Seventy-five percent (*n* = 160) of the patients had CD4+ ≤ 50 cells/μL and 25.2% (*n* = 54) had > 50 cells/μL.

An overall 51.0% (*n* = 194) of the patients in this study were on ART at diagnosis of CMVR. Only two patients did not have records regarding their ART status at the diagnosis of CMVR. Of the 194 patients on ART, records of 188 (96.9%) indicated whether they were on ART for at least 6 months. There was a higher proportion of patients on ART ≤ 6 months, *n* = 119 (63.3%), compared with those on ART > 6 months, *n* = 69 (36.7%): using the *z*-test the difference was statistically significant, *P* < 0.01. Of the 69 patients on ART for > 6 months, 20 (30%) self-reported ART ‘issues’, including non-adherence and regimen changes by their HIV healthcare facility. Eight patients (6.7%) on ART ≤ 6 months self-reported ART issues. A total of 187 (49%) patients were ART naive at diagnosis of CMVR. In this study, a total of 306 (81.6%) patients were either ART naïve or on ART for ≤ 6 months.

A total of 320 patients had documentation of whether they were on TB treatment at the time of diagnosis of CMVR and 116 (36.3%) of them were on TB treatment at diagnosis. Three hundred and twenty-two patients had records regarding prior TB and 105 (32.6%) patients reported that they had been previously treated for TB. Eight patients had been on TB treatment both before and at their diagnosis of CMVR. Of the 194 patients on ART at diagnosis, 162 (83.5%) patients had data regarding whether they were on TB treatment at diagnosis of CMVR and a total of 55 (34%) patients were on ART and TB treatment at diagnosis. Patients on TB treatment at diagnosis of CMVR were not more likely to default anti-CMVR therapy (RR: 1.02; 95% CI: 0.799–1.302; *P* = 0.437).

## Discussion

The distribution of patients in this study is similar to the ethnic, age and sex distribution of PLWH in SA.^26^ In SA, HIV infection is found to be more prevalent in black African people, peaks between 35 years and 39 years and is 15.7% higher in women.^26^ The study from WC also reported a similar age and gender distribution.^25^ This differs from the CMVR subset of LSOCA whose participants were 44% white American people, 91% greater than 35 years old and 80.4% male.^6^ And demonstrates the difference in the distribution of PLWH and CMVR in SA, in comparison with the developed world. The female predominance could reflect access to HIV healthcare antenatally, which may have allowed for referrals to ophthalmic services for visual loss complaints. The median CD4+ count at diagnosis of CMVR in this study (22 cells/μL) and that reported by LSOCA (28 cells/μL) were similar.^5^ The higher proportion of bilateral disease (64.3%) reported in this study in comparison with LSOCA (31.3%) suggests that patients were diagnosed a substantial period after the initial development of retinal lesions.^5,20^ The WC study reported a 72.5% proportion of patients on ART at diagnosis of CMVR, whilst 77.9% of the patients in LSOCA were on ART at enrolment.^6,25^ This study reports an overall 51% of patients on ART at diagnosis of CMVR. In this study, 36.3% of the participants were on TB treatment at diagnosis and 32.6% already received TB treatment prior to their diagnosis of CMVR. A previous study from KZN reported that 56.6% of their patients with CMVR either had current or prior TB.^24^

Despite the introduction of ART in April 2004, the annual number of new cases of CMVR initially increased to a peak in 2006. The reason for this increase is likely multifactorial. It possibly reflects a delay in initiating ART despite availability amongst PLWH with a CD4+ count ≤ 200 cells/µL. Furthermore, prior to ART there was inadequate access to HIV healthcare in the province, heightened stigma, and a lack of HIV clinicians and nurses. This could have impacted the number of referrals to ophthalmic care. The diagnosis of CMVR is only possible when there are sufficient healthcare resources to prevent mortality from other opportunistic infections, such as TB and pneumocystis carinii pneumonia, which occur at higher CD4+ counts, the so-called ‘survival bias’.^1,20,24^ Since ART became available in 2004, HIV became a chronic disease and the prevalence of PLWH with lower CD4+ counts increased, as well as the number of patients presenting to HIV clinicians for ART and subsequent referrals to ophthalmology, with a subsequent increase in the diagnosis of CMVR. Immune recovery retinitis (unmasking or paradoxical CMVR) is unlikely to be related to the initial increase in CMVR found in this study, as IRR is found to occur in patients with CD4 > 100 cells/μL, and the median CD4+ cell count in this study was 22 cells/μL.^23^ Delaying the initiation of ART in asymptomatic patients until the CD4 count decreased to 200 cells/μL was not sufficient enough to prevent CMVR. Once ART was offered to all PLWH with a CD4+ count ≤ 350 cells/μL in April 2012, there was a statistically significant decrease (61.76%, *P* = 0.001) in the average number of CMVR cases/year. This is in accordance with the reported decline in PLWH in KZN at risk for CMVR with severe immune impairment: between 2010–2011 and 2014–2015, there was a decrease in the average proportion of CD4+ samples, with a CD4+ count < 100 cells/μL from 11.2% to 7.3%.^27^ The documented decrease in the burden of CMVR over time shown in this study is in contrast with a previous review of reported data on trends in CMVR from developing countries, which concluded that the prevalence was similar for the period 1993–2002 and 2009–2013.^20^ With regard to developed countries, LSOCA observed a 90% reduction in the incidence of CMVR in the ART era,^4,5,6,7^ and similar results were reported by studies from Germany^28^ and Austria.^29^

In addition to progressive ART CD4+ count initiation criteria and subsequent expanded ART coverage, the incidence of HIV in SA decreased by 55% over the 2010–2019 period, with the decline being greatest in KZN (61%).^3^ In 2017, SA reached the first of the UNAIDS 90-90-90 targets^8^ and laid out a National Strategic Plan (NSP) to meet the remaining targets by 2022.^9,14^ eThekwini had made the greatest progress towards these targets in SA, with an estimated 92-73-91 in comparison with the national estimates of 91-68-86 in 2018.^15^ Despite this improvement, the decrease in the number of newly diagnosed CMVR per year levelled off after 2011. A similar plateau was found in developed countries,^4,5,6,7^ and is thought to be related to late testing and initiation of ART.^1,4,20^ This was highlighted in this study by the large proportion (81.6%) of patients who were either ART naive at the diagnosis of CMVR in the ART era or had been initiated on ART ≤ 6 months prior.

The study also re-iterates that patients on ART for ≤ 6 months remain at risk for CMVR, which has been previously reported.^4,5,6^ This could be as a result of persistent low CD4 counts in the first 6 months of ART, as well as the known 6-month delay in developing CMV-specific immunity following ART.^22^ Co-management between ophthalmologists and HIV clinicians is vital for the patients on ART. Whilst it is beyond the scope of this study to comment on ART failure, it is clear from the number of patients who self-reported ART disturbances that ART failure is likely a contributing factor to the occurrence of CMVR, particularly in patients on ART for > 6 months.

With regard to the anti-CMV therapy, oral options, such as valganciclovir, are preferred.^4,5,6,7,20,25^ However, a cost analysis carried out in 2010 for SA showed that oral valganciclovir would cost R19 479.32 for 21 days per patient.^30^ Treatment in developing countries, therefore, involved regular ‘off-label’ intravitreal injections of the cheaper ganciclovir.^20,24,25^ In July 1996, the ophthalmic unit managing CMVR in this study was the first to administer intravitreal anti-CMVR therapy in SA.^24^ This resulted in a significant burden on ophthalmology services at the study location, particularly in the period prior to April 2012. In 2013, Roche and the Medicines Patent Pool first signed an agreement that made oral valganciclovir available at a cost reduction of up to 90% in low- and middle-income countries.^31^ Subsequently, valganciclovir was added to the essential medicines list (EML) of the World Health Organization and the fourth edition of the SA hospital level standard treatment guidelines (STGs) and EML in 2015.^32,33^ The availability at provincial level is, therefore, possible following approval by provincial pharmacy and therapeutics committees. Following the improved access to valganciclovir, as well as the significant proportion of patients who defaulted intravitreal ganciclovir in this study, the authors recommend transitioning to oral therapy and intravitreal augmentation as necessary. This has already begun at other ophthalmic centres managing CMVR in SA.^21^ This will improve adherence to treatment, treat systemic CMV that is common in bilateral CMVR and decrease mortality, even in patients on ART.^4,5,6,7,20^ It will also allow treatment to be initiated by trained HIV clinicians in the community, particularly for those patients who cannot present to ophthalmologists based in tertiary referral centres. Because of the considerable number of cases of atypical and co-existent retinitis reported in this study and known complications of CMVR, close collaboration will be needed between HIV and ophthalmic services.

Screening for CMVR by community-based HIV practitioners has been shown to be as effective as screening by ophthalmologists.^17^ Retinal necrosis because of CMVR is irreversible, and it may already be widespread by the time the patient becomes symptomatic. Active CMV lesions can be detected by indirect fundoscopy in up to 30% of asymptomatic PLWH.^7^ Efforts have, therefore, been made globally to train groups of HIV clinicians to screen for CMVR.^34^ Recently, the group from Myanmar was the first to report the use of oral valganciclovir by HIV clinicians for treating CMVR in a developing country, and concluded with the finding that oral valganciclovir was safe and effective.^35^ To our knowledge, there has been no effort to train HIV clinicians to screen for CMVR in SA. Currently, there is also no consensus regarding screening by ophthalmologists, particularly in the ART era.^19^ In order to further eliminate visual loss from CMVR, the authors recommend the development of a CMVR screening programme in SA.

This study is not without limitations. There is no CMVR screening system in place for PLWH, and patients were referred to us with a provisional diagnosis resulting in a referral bias. We therefore did not have access to controls and cannot comment on the incidence of CMVR in all PLWH in eThekwini or possible risk factors. The potential for human error when entering ICD-10 codes for patients could also have resulted in many patients being overlooked. Because of the retrospective nature of this study, information was sometimes missing, and the study was limited to the variables available. Details of the ART regimens, the time since HIV diagnosis and CD4+ count at diagnosis of HIV were not available. HIV viral loads were not requested by the ophthalmologists managing the patients who reported ART disturbances, and, therefore, we cannot comment on ART failure. Adherence with ART or the absence of a change in the ART regimen was not specifically documented in all patients on ART, and we therefore cannot make comparisons with the number of patients who self-reported ART disturbances. Oral valganciclovir was not offered to participants in this study because of the lack of awareness of its inclusion in the SA STG and EML. Both IRU and IRR cannot be excluded retrospectively and are therefore not reported.

## Conclusion

Antiretroviral treatment has resulted in a clear decrease in the burden of CMVR on ophthalmic services with time, particularly after ART was offered to PLWH with a CD4 count ≤ 350 cells/µL. However, there remains a population at risk for CMVR, and therefore further collaboration between ophthalmic services managing CMVR and HIV clinicians is necessary. Strategies to further eliminate visual loss from CMVR should include training HIV clinicians to screen for CMVR in patients at risk, which includes those initiated on ART late with a CD4+ count < 100 cells/μL and should continue until ART has been maintained for at least 6 months. Those diagnosed with CMVR despite ART for > 6 months need to be prioritised for assessment of ART failure by HIV clinicians. Commencing oral valganciclovir prior to referral to ophthalmic care can be considered. The transition to oral valganciclovir and augmentation with intravitreal ganciclovir as necessary by all ophthalmic services managing CMVR in the country is also recommended. This study spanned 15 years and allowed for data collection regarding the burden of CMVR for a large proportion of the HIV-endemic, KZN province in South Africa. This is yet to be achieved for comparable opportunistic infections.
